# Impact of the Introduction of Calcimimetics on Timing of Parathyroidectomy in Secondary and Tertiary Hyperparathyroidism

**DOI:** 10.1245/s10434-016-5450-6

**Published:** 2016-07-26

**Authors:** Willemijn Y. van der Plas, Anton F. Engelsman, Akin Özyilmaz, Anouk N. van der Horst-Schrivers, Kornelis Meijer, Gooitzen M. van Dam, Robert A. Pol, Martin H. de Borst, Schelto Kruijff

**Affiliations:** 1 Department of Surgery, University Medical Center Groningen, University of Groningen, Groningen, The Netherlands; 2 Department of Surgery, Academic Medical Center, Amsterdam, The Netherlands; 3 Department of Nephrology, University Medical Center Groningen, University of Groningen, Groningen, The Netherlands; 4 Department of Endocrinology, University Medical Center Groningen, University of Groningen, Groningen, The Netherlands; 5 Department of Clinical Chemistry, University Medical Center Groningen, University of Groningen, Groningen, The Netherlands; 6 Department of Nuclear Medicine and Molecular Imaging and Intensive Care, University Medical Center Groningen, University of Groningen, Groningen, The Netherlands; 7 Dialysis Center Groningen, Groningen, The Netherlands

## Abstract

**Background:**

Hyperparathyroidism (HPT), both secondary and tertiary, is common in patients with end-stage renal disease, and is associated with severe bone disorders, cardiovascular complications, and increased mortality. Since the introduction of calcimimetics in 2004, treatment of HPT has shifted from surgery to predominantly medical therapy.

**Objective:**

The aim of this study was to evaluate the impact of this change of management on the HPT patient population before undergoing (sub-)total parathyroidectomy (PTx).

**Methods:**

Overall, 119 patients with secondary or tertiary HPT undergoing PTx were included in a retrospective, single-center cohort. Group A, who underwent PTx before January 2005, was compared with group B, who underwent PTx after January 2005. Patient characteristics, time interval between HPT diagnosis and PTx, and postoperative complications were compared.

**Results:**

Group A comprised 70 (58.8 %) patients and group B comprised 49 (41.2 %) patients. The median interval between HPT diagnosis and PTx was 27 (interquartile range [IQR] 12.5–48.0) and 49 (IQR 21.0–75.0) months for group A and B, respectively (*p* = 0.007). Baseline characteristics were similar among both groups. The median preoperative serum parathyroid hormone (PTH) level was 936 pg/mL (IQR 600–1273) for group A versus 1091 pg/mL (IQR 482–1373) for group B (*p* = 0.38). PTx resulted in a dramatic PTH reduction (less than twofold the upper limit: A, 80.0 %; B, 85.4 %), and postoperative complication rates were low in both groups (A: 7.8 %; B: 10.2 %) [*p* = 0.66].

**Conclusions:**

The introduction of calcimimetics in 2004 is associated with a significant 2-year delay of surgery with continuously elevated preoperative PTH levels, while parathyroid surgery, even in a fragile population, is considered a safe and effective procedure.

Hyperparathyroidism (HPT) is a common complication in patients with end-stage renal disease (ESRD).^1^^,^ 2 In chronic kidney disease (CKD), calcium and phosphate homeostasis are progressively deregulated, resulting in CKD-related mineral and bone disorders (CKD-MBD), commonly accompanied by secondary HPT.^3^^,^ 4 Tertiary HPT develops when hyperplastic parathyroid glands no longer respond to the plasma calcium concentration and function autonomously, which is clinically most evident when HPT does not resolve after successful kidney transplantation (KTx).^5^^,^ 6 Both secondary and tertiary HPT are associated with cardiovascular complications and increased mortality.^7^^–^ 10

More than a decade ago, the main treatment options for HPT consisted of calcium-containing phosphate binders, vitamin D sterols and (sub-)total parathyroidectomy (PTx).^11^ More recently, recommended strategies include the use of (primarily) non-calcium-containing phosphate binders, vitamin D analogs, or a combination of these to decrease parathyroid hormone (PTH) levels.^12^ PTx is currently only recommended in patients with severe HPT who fail to respond to medical treatment.^12^ Since its introduction in 2004, the calcimimetic agent cinacalcet has become a common first-line therapy for HPT patients insufficiently responsive to vitamin D and phosphate binders. Despite the lack of randomized studies that directly compare cinacalcet with PTx, the introduction of cinacalcet seems to have led to a change in treatment strategy and, consequently, PTx is less often performed.^13^ Despite this policy change, several questions have been raised about the efficacy, side effect profile, and costs of cinacalcet.^14^^,^ 15 Moreover, studies evaluating the effect of cinacalcet on lowering PTH levels show contradictory results.^7^^,^ 14 ^,^ 16 A recent Cochrane review, which was strongly driven by the primary results from the EVOLVE trial, concluded that there is no clear evidence that cinacalcet reduces the risk of death or major cardiovascular events.^16^^,^ 17 Consequently, cinacalcet is no longer subsidized by the Australian Government.^18^ It is unclear how the introduction of cinacalcet affected the secondary and tertiary HPT patient population ultimately requiring PTx. To address this, we performed a retrospective, single-center observational study to compare PTx patient characteristics, time from diagnosis to surgery, and PTx efficacy and safety outcomes before and after the introduction of calcimimetics.

## Materials and Methods

### Study Population

The study population of this retrospective, single-center study consisted of all ESRD patients with secondary or tertiary HPT who underwent PTx and were aged 18 years and older at the time of surgery at the University Medical Center Groningen (UMCG), The Netherlands, between 1991 and 2015. Patients were excluded if they had (para-)thyroid malignancy in their medical history and/or previous surgery in the neck area. Data of the included patients were extracted from the hospital’s electronic patient record system.

This study was approved by the local Medical Ethical Committee (METc 2015/339), and patient data were processed and electronically stored according to the declaration of Helsinki Ethical principles for medical research involving human subjects.

### Study Design, Primary and Secondary Endpoints

Patients were divided into two groups according to date of surgery: before (group A) or after (group B) January 2005, as cinacalcet was introduced in The Netherlands in 2005.

The primary outcome measure was time from HPT diagnosis to PTx. Date of HPT diagnosis was defined as the moment vitamin D supplements were first prescribed to suppress PTH overproduction. Furthermore, we compared patient characteristics prior to surgery, including age, sex, American Society of Anesthesiologists (ASA) physical status classification, body mass index (BMI), history of diabetes mellitus (DM) according to the American Diabetes Association (ADA) 2010 criteria,^19^ time on dialysis, type of PTx, use of vitamin D analogs, phosphate binders and cinacalcet, and a history of KTx. Furthermore, laboratory values (calcium, phosphate, albumin, alkaline phosphatase and PTH) and 30-day postoperative complications were recorded. The serum calcium level was adjusted for albumin according to the following formula: adjusted total calcium (mmol/L) = measured calcium (mmol/L) + (0.025 * (40 – [albumin (g/L)]). Reference values were 2.20–2.60 mmol/L. From 1991 until 2006, PTH analysis was performed using the PTH-intact assay from Nichols Institute Diagnostics (San Juan Capistrano, CA, USA). In this period, several assays have been deployed in our patients, using the same antibodies with different detection methods (radioimmunoassay and chemiluminescent immunoassays). Since February 2006, PTH has been analyzed using PTH-intact assays using the Immulite 2500 (Siemens Healthcare Diagnostics, Deerfield, IL, USA) and the Cobas e601 immunology analyzer (Roche Diagnostics, Mannheim, Germany). In-house comparison of consecutive PTH assays showed only significant deviation between the Nichols Advantage ILMA and the Siemens Immulite 2500. To compare data before and after the method conversion, the data before 2006 were recalculated using the following conversion factor: Immulite (pmol/L) = 1.27 × Advantage (pmol/L) + 0.5.^20^^–^ 22 Reference values for PTH were 16–87 pg/mL or 1.8–9.6 pmol/L.

Persistent postoperative hypocalcemia was defined as the need for calcium supplements 6 months after PTx. Information about weight of the removed parathyroid glands was extracted from pathology reports; the weight of the largest removed parathyroid gland was used for comparative analyses.

### Statistical Analysis

Descriptive tests were used to express continuous variables as mean ± standard deviation (SD) or median with interquartile range (IQR), and categorical variables were described as count (*n*) and percentage (%). Patient characteristics were compared using the independent sample *t*-test or Mann–Whitney *U*-test for continuous variables, and differences between nominal variables were determined using the Pearson Chi-square test. Distribution was assessed using the Shapiro–Wilk normality test. *p*-Values <0.05 were considered statistically significant. Statistical analysis was performed using SPSS Statistics version 22.0 (IBM Corporation, Armonk, NY, USA.)

## Results

### Study Population

**Fig. 1:**
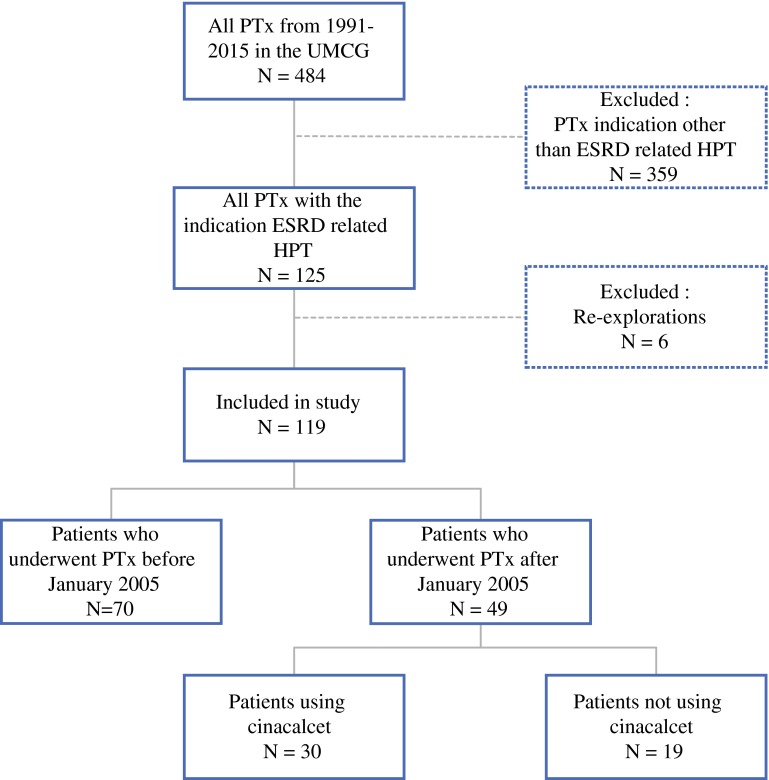
Cohort patient selection process. *PTx* parathyroidectomy, *UMCG* University Medical Center Groningen, *ESRD* end-stage renal disease, *HPT* hyperparathyroidism

Between 1991 and 2015, a total of 484 PTxs were performed in our center. After applying the aforementioned inclusion and exclusion criteria, a total of 119 (24.6 %) patients were included in the study (Fig. 1).

### Patient Characteristics

**Fig. 2:**
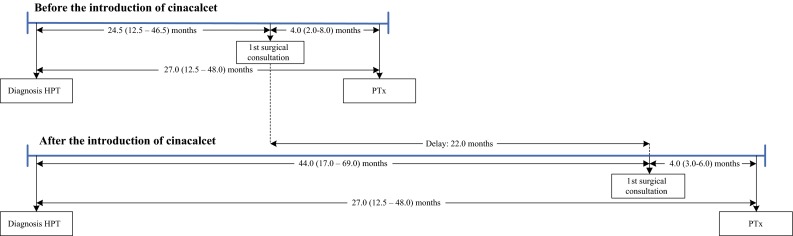
Timeline from diagnosis of hyperparathyroidism until parathyroidectomy. *HPT* hyperparathyroidism

Patient characteristics at PTx are listed in Table 1. Seventy patients (58.8 %) underwent PTx before the introduction of cinacalcet (group A) and 49 (41.2 %) patients underwent PTx after the introduction of cinacalcet (group B). Median age of all patients was 53.0 years (IQR 40–60), 62.2 % were women, and 82.4 % of all patients were classified as ASA III or higher. At the time of PTx, 77.9 % of all patients received vitamin D analogs and/or phosphate binders (63.7 % received vitamin D supplements and 61.9 % received phosphate binders). After January 2005, 88.9 % of the prescribed phosphate binders were non-calcium-containing versus 41.2 % before January 2005 (*p* *<* 0.001). In group B, 30 patients (61.2 %) were using cinacalcet at the time of PTx. There was no significant difference in the number of parathyroid surgeries per year before versus after 2005 [median (IQR) 4.0 (2.0–9.0) vs. 4.0 (2.0–5.0); *p* *=* 0.65). The median interval between HPT diagnosis and PTx was 27 months (12–48) for group A and 49 months (21.0–75.0) for group B (*p* *=* 0.007). A diagram illustrating the delay from diagnosis of HPT until PTx after 2005 is presented in Fig. 2.

**Table 1:** Patient characteristics before parathyroidectomy

Characteristic	Overall [*N* = 119]	Group A—before the introduction of cinacalcet [*N* = 70]	Group B—after the introduction of cinacalcet [*N* = 49]	*p*-Value
Age at surgery, years	53.0 (40.0–60.0)	52.5 (40.8–60.0)	54.0 (38.5–59.5)	0.81
Sex, female	74 (62.2)	47 (67.1)	27 (55.1)	0.18
BMI, kg/m^2^	24.3 (21.4–27.1)	23.4 (20.7–25.8)	25.3 (23.3–27.3)	0.05
History of diabetes				0.21
Type I	3 (2.5)	1 (1.4)	2 (4.1)	
Type II	11 (9.2)	8 (11.4)	3 (6.1)	
Steroid-induced diabetes	2 (1.7)	0 (0)	2 (4.1)	
ASA classification				0.12
II	21 (17.6)	9 (12.9)	12 (24.5)	
III	97 (81.5)	61 (87.1)	36 (73.5)	
IV	1 (0.8)	0 (0)	1 (2.0)	
History of KTx	21 (17.9)	11 (15.7)	10 (21.3)	0.66
Receiving dialysis	90 (76.3)	57 (81.4)	33 (68.8)	0.11
Duration of dialysis, months	46.0 (24.0–76.0)	46.0 (28.0–78.0)	48.5 (21.5–76.0)	0.67
Use of vitamin D analogs	72 (63.7)	38 (59.4)	34 (69.4)	0.27
Use of phosphate binders	70 (61.9)	34 (53.1)	36 (73.5)	0.03
Non-calcium-containing	46 (65.7)	14 (41.2)	32 (88.9)	<0.001
Use of cinacalcet	30.0 (25.2)	0 (0.0)	30 (61.2)	<0.001
Time interval from HPT diagnosis to PTx, months	33.5 (16.8–56.3)	27.0 (12.5–48.0)	49.0 (21.0–75.0)	0.007

Data are expressed as median (interquartile range) or *n* (%)

*BMI* body mass index, *ASA* American Society of Anaesthesiologists, *KTx* kidney transplantation, *HPT* hyperparathyroidism, *PTx* parathyroidectomy

### Biochemistry

Preoperative laboratory values are listed in Table 2. PTH levels were strongly elevated in both groups [median (IQR) 936 pg/mL (600–1273) and 1091 pg/mL (482–1373) for groups A and B respectively; *p* *=* 0.38). Mean corrected calcium level was 2.60 ± 0.34 mmol/L, and was significantly different between the two groups (*p* *=* 0.008)

**Table 2:** Preoperative laboratory variables

	Overall [*N* = 119]	Group A—before the introduction of cinacalcet [*N* = 70]	Group B—after the introduction of cinacalcet [*N* = 49]	*p*-Value
PTH, pg/mL	963.6 (527.3–1300.0)	936.4 (600.0–1272.7)	1091.0 [482.2-1372.8]	0.38
Corrected calcium, mmol/L	2.60 ± 0.34	2.67 ± 0.36	2.50 ± 0.28	0.008
Phosphorus, mmol/L	1.59 ± 0.58	1.67 ± 0.56	1.49 ± 0.60	0.09
Alkaline phosphatase, U/L	147.5 (99.25–203.75)	150.5 (100.5–226.0)	137.0 [94.0-194.5]	0.48
Calcium-phosphorus product, mmol^2^/L^2^	4.06 (2.98–5.19)	4.34 (3.27–5.82)	3.47 (2.55–4.74)	0.01

Data are expressed as mean ± SD or median (interquartile range)

*PTH* parathyroid hormone

**Fig. 3:**
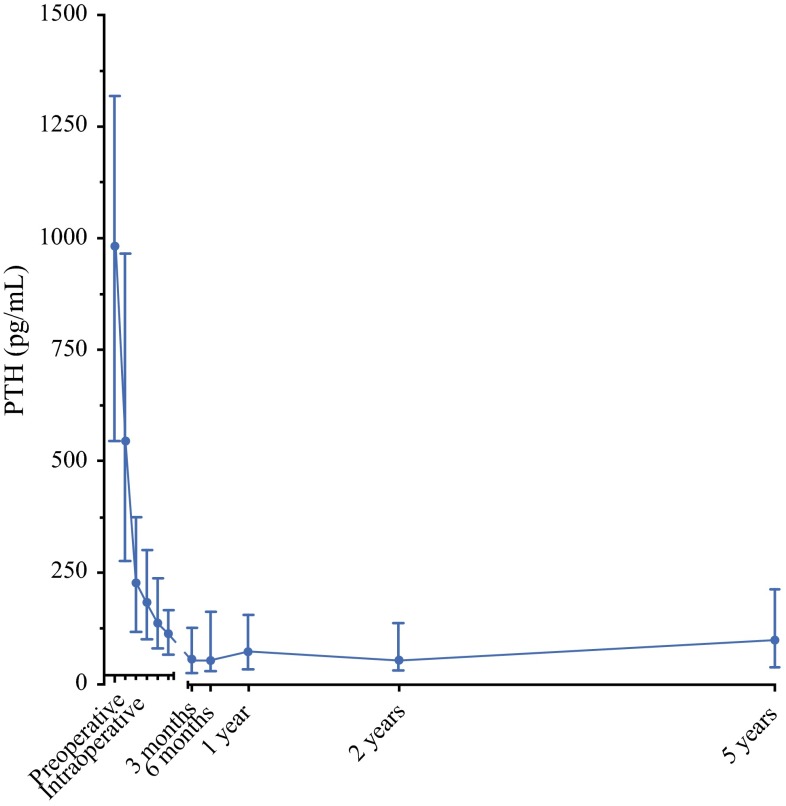
Median preoperative, intraoperative and postoperative PTH levels of 119 patients undergoing parathyroidectomy. *PTH* parathyroid hormone

Median preoperative, intraoperative and postoperative PTH levels are shown in Fig. 3. PTH levels decreased significantly after PTx: at 3 months after PTx the median PTH reduction from baseline was 96.0 % (84.6–99.1). Overall, at 3 months after PTx, 82.4 % of all patients had PTH levels below 162 pg/mL (two times the upper reference limit, acceptable according to the Kidney Disease: Improving Global Outcomes [KDIGO] guidelines). Patients in groups A and B had postoperative PTH levels of 45.0 pg/mL (9.6–152.3) and 20.5 pg/mL (2.5–94.6), respectively. Postoperative PTH levels were not significantly different (*p* *=* 0.079). Although PTH levels slightly increased at 5 years after PTx [37.3 pg/mL (6.8–107.7) at 3 months postoperatively vs. 80.5 pg/mL (19.7–193.2) at 5 years postoperatively; *p* *=* 0.04], 68.2 % of all patients still had PTH levels below 162 pg/mL.

### Parathyroid Gland Weight

Median weight of the largest resected parathyroid gland was 1.16 g (0.70–1.86). No significant difference in parathyroid gland weight was observed between groups A and B [median (IQR) 1.11 g (0.63–1.82) vs. 1.28 g (0.72–1.90), respectively; *p* *=* 0.51).

### Re-Exploration Rate

Six patients (5.0 %) required re-exploration. In four cases, persistent HPT was the indication for re-exploration, while the two remaining patients had recurrent HPT. Five of six patients who underwent re-exploration underwent subtotal PTx, and one patient underwent total PTx with autotransplantation. In study group A, re-exploration was required in five (7.1 %) cases, while one (2.0 %) patient in group B underwent reoperation. The difference in re-exploration rate between the two groups did not reach statistical significance (*p* *=* 0.21). Median time from initial PTx to re-exploration was 23.5 months (4–68).

### Postoperative Complications

Postoperative complications are listed in Table 3. In all patients, the number of complications, including mortality, recurrent laryngeal nerve damage, surgical site problems (SSP), pneumonia, and intensive care unit (ICU) admission was 10 in total (8.8 %). Only one (0.8 %) patient presented with postoperative wound bleeding that required reoperation. Other wound problems were minimal and comprised minute bleeding or minor infection requiring antibiotics. No significant difference in the number of complications was observed between the two groups (*p* *=* 0.66).

**Table 3:** Postoperative complications

	Overall [*N* = 119]	Group A—before the introduction of cinacalcet [*N* = 70]	Group B—after the introduction of cinacalcet [*N* = 49]	*p*-Value
Mortality	1 (0.8)	0 (0.0)	1 (2.0)	0.23
Recurrent laryngeal nerve damage	2 (1.8)	0 (0.0)	2 (4.1)	0.10
Surgical site problems	4 (3.5)	3 (4.7)	1 (2.0)	0.45
Pneumonia	2 (1.8)	2 (3.1)	0 (0.0)	0.21
ICU admission	1 (0.8)	0 (0.0)	1 (2.0)	0.25
Calcium supplements 6 months postoperatively	45 (39.8)	27 (42.2)	18 (36.7)	0.56

Data are expressed as *n* (%)

*ICU* intensive care unit

## Discussion

This single-center, retrospective study documents a significant 22-month delay from diagnosis to (sub-)total PTx in patients with HPT since the introduction of the calcimimetic agent cinacalcet in 2005. Moreover, we observed that, even after the introduction of cinacalcet, median preoperative PTH levels have remained unchanged. Our findings raise questions about the efficacy of current treatment of patients with HPT and the consequently prolonged exposure to high PTH levels. To our knowledge, this is the first study documenting the difference in time interval between HPT diagnosis and parathyroid surgery since cinacalcet became available.

Several reasons may underlie the 22-month referral delay. First, in the past decade, there has been an increasing interest for the medical treatment of HPT, particularly with cinacalcet.^23^ The availability of cinacalcet seems to have contributed to a strategy change intending to delay surgery as long as medically possible, and preferably until KTx, which might result in resolution of HPT.^5^ Indeed, several studies have aimed to reduce the incidence of PTx.^13^^,^ 16 ^,^ 24 However, whether delaying surgery with long-term medical therapy is truly beneficial for the individual patient is unknown. While waiting for KTx, HPT often becomes refractory, eventually leading to an unavoidable PTx.^23^ Eventually, approximately 30 % of transplanted patients have ongoing (tertiary) HPT.^6^ When calcium-phosphate homeostasis does not normalize after KTx, medical treatment often delays definite surgical intervention, as Lou et al. concluded that PTx is underused in patients with tertiary HPT.^5^^,^ 6 Therefore, we need predictive factors for developing refractory or tertiary HPT to determine in advance which patients will eventually need PTx after transplantation. In The Netherlands, the Dutch Hyperparathyroid Study Group (DHSG), a multicenter initiative, is currently trying to answer these questions in larger retrospective patient data sets.^25^ A second reason for the observed referral delay may be that standardized and specific indications for referral for surgery are not available.^5^^,^ 23 The overall change in guidelines of HPT management might be another explanation for the late referral. The 2003 Kidney Disease Outcomes Quality Initiative (K/DOQI) guidelines recommended maintaining PTH values below 300 pg/mL (three to five times the upper limit), whereas the 2009 KDIGO guidelines recommended PTH levels between two and nine times the upper limit (up to 746 pg/mL; grade 2C recommendation) 11 ^,^ 12; however, little evidence is available to support these (changes in) guidelines. Lastly, group B received predominantly non-calcium-containing phosphate binders as opposed to group A patients, who mainly received calcium-containing phosphate binders. This prescription change might have also led to a referral delay as patients using calcium-containing medication might be referred in an earlier stage because of high serum calcium levels.

This study was not designed to conclude whether patients would benefit from earlier surgery; however, with the persistence of long-term elevated PTH levels in ESRD patients, several problems may arise. Tentori et al. found a positive correlation between elevated PTH levels and cardiovascular, and all-cause mortality and increased cardiovascular hospitalization.^2^ Although data on the effects of long-term elevated PTH levels on kidney (graft) function are limited, a large post hoc analysis showed a significant association between tertiary HPT and adverse graft outcome after KTx.^26^ In addition, long-term conservative treatment using vitamin D derivatives, phosphate binders and calcimimetics entails high costs.^17^^,^ 27 A cost utility analysis showed that PTx is less expensive and more cost effective at 7.25 months in comparison to cinacalcet-based medical therapy.^15^ Finally, the EVOLVE trial revealed a high incidence of adverse effects accompanying cinacalcet use (adverse effects such as vomiting and nausea were reported at 45.9 % in the cinacalcet group vs. 18.9 % in placebo group), often leading to discontinuation of the drug.^16^

Even after a 22-month delay in this very fragile population (ASA III or even IV), parathyroid surgery led to low complication rates and an effective decrease in PTH levels. It could be that the potential adverse effects of the delayed referral are balanced by the improved quality of parathyroid surgery with the use of less invasive surgical procedures, concentration of care in specialized centers, heat sealing devices, and improved imaging for preoperative localization.^28^^,^ 29

Our results regarding safety are at variance with a nationwide US study showing much higher complication rates, supporting the need to concentrate PTx procedures in higher-volume, specialized centers.^30^ Moreover, our results are in line with other previous studies demonstrating much lower complication rates, including mortality, recurrent laryngeal nerve damage, SSP, and ICU admission occurring at <10 %.^5^^,^ 31 ^–^ 34 Although not significant, there were less reoperations in the group after the introduction of cinacalcet. With our current data, we were not able to conclude whether there is a relationship between the use of cinacalcet and the need for re-explorations.

This study has certain limitations that need to be addressed. First, because of its retrospective nature, our data may be biased by variations in the recording methods used in our electronic patient record systems, and patients lost to follow-up. Second, our results may have limited generalizability as they were from a single-center study. Our study was not designed to compare PTx with cinacalcet. Preparations for a large, multicenter, randomized control trial comparing PTx and cinacalcet with long-term follow-up to define the treatment of choice in patients with chronic renal failure are currently underway in The Netherlands (RHINO trial).

## Conclusions

The introduction of cinacalcet is associated with a 22-month delay of surgical treatment of HPT. Since the introduction of calcimimetics, we have not recorded lower preoperative PTH levels. On the other hand, parathyroid surgery, even in a fragile population, is considered both an effective and safe procedure.
